# Enhancement of gastrointestinal anastomosis healing via a small intestinal submucosa bio-patch: modulating IL-22 secretion by type 3 innate lymphoid cells and microbial structures

**DOI:** 10.3389/fbioe.2026.1752619

**Published:** 2026-02-13

**Authors:** Hao-Jie Zhong, Yuan Zhou, Jia-Wen Zhao, Wei-Ran Chen, Nai-Yang Zhan, Yong-Qiang Zhan

**Affiliations:** 1 Department of Hepatobiliary and Pancreatic Surgery, The First Affiliated Hospital of Shenzhen University, Shenzhen Second People’s Hospital, Shenzhen, China; 2 Department of Rheumatology, Shenzhen Traditional Chinese Medicine Hospital, The Fourth Clinical Medical College of Guangzhou University of Chinese Medicine, Shenzhen, China; 3 Phamaceutical Department, Hubei Cancer Hospital, Tongji Medical College, Huazhong University of Science and Technology, Wuhan, China; 4 Department of Gastroenterology, The First Affiliated Hospital of Guangdong Pharmaceutical University, Guangzhou, China

**Keywords:** fungi, gastrointestinal anastomosis, metabolites, microbiota, small intestinal submucosa bio-patch, type 3 innate lymphoid cells

## Abstract

**Purpose:**

Anastomotic leakage and impaired healing remain major complications in gastrointestinal (GI) surgery. Small intestinal submucosa (SIS), a biological scaffold, has shown regenerative potential but its mechanisms in GI anastomotic healing remain unclear. This study aimed to investigate the effects of an SIS bio-patch on intestinal anastomotic healing, focusing on immune modulation, microbiota reshaping, and metabolic changes.

**Methods:**

C57BL/6 mice underwent GI anastomosis with or without SIS bio-patch implantation. Five days post-operation, tissues were collected for histology, immunofluorescence, flow cytometry, 16S and ITS sequencing, and untargeted metabolomics. Immune cell composition, barrier protein expression, microbiota composition, and metabolic signatures were analyzed.

**Results:**

SIS bio-patch significantly reduced inflammation and enhanced mucosal barrier integrity, as evidenced by reduced TNF-α and IL-6 and increased ZO-1 and occludin expression. SIS increased IL-22^+^ILC3s (type 3 innate lymphoid cells) and decreased the Th17/Treg ratio without altering macrophage polarization. Microbiota analysis showed increased abundance of *Bifidobacterium* and *Alloprevotella*, correlating positively with IL-22^+^ILC3s. Fungal sequencing revealed higher *Fungi gen.* Incertae sedis levels, associated with beneficial immune profiles. Metabolomics showed elevated amino acids and biotin metabolism in SIS-treated tissues, which may support epithelial regeneration.

**Conclusion:**

SIS bio-patch promotes anastomotic healing by enhancing IL-22^+^ILC3-mediated repair, rebalancing adaptive immunity, reshaping microbial communities, and upregulating pro-regenerative metabolic pathways. These findings support the use of SIS as an immunomodulatory biomaterial for gastrointestinal repair.

## 1 Introduction

Gastrointestinal anastomosis is a fundamental surgical procedure used to restore intestinal continuity following tumor resection, traumatic injury, or bowel resection in inflammatory bowel diseases (Marrache et al., 2021). Despite continuous advances in surgical techniques and perioperative management, anastomotic failure remains a major clinical challenge. Complications such as anastomotic leakage, stricture formation, and postoperative bleeding substantially increase morbidity and mortality, prolong hospital stay, and worsen long-term outcomes (Li et al., 2023; Velotti et al., 2021). Therefore, developing effective strategies to enhance anastomotic healing remains a critical unmet need in gastrointestinal surgery.

In recent years, biologically derived scaffolds have attracted increasing attention for their potential to promote tissue repair. Among these, small intestinal submucosa (SIS), an extracellular matrix–based biomaterial derived from porcine intestine, exhibits excellent biocompatibility, low immunogenicity, and favorable mechanical properties (Zhang et al., 2023; Zhao et al., 2024). SIS has been widely applied in vascular reconstruction, abdominal wall repair, wound healing, and urological surgery, with encouraging clinical outcomes (Ying et al., 2024; Zhao et al., 2024). In gastrointestinal surgery, SIS has been shown to mechanically reinforce anastomoses and improve early sealing integrity (Hoeppner et al., 2010). However, most existing studies have primarily focused on its structural or biomechanical advantages, while the biological mechanisms by which SIS influences local immune responses and the intestinal microenvironment during anastomotic healing remain insufficiently explored.

The immune system within the gastrointestinal tract plays a central role in maintaining intestinal homeostasis and facilitating tissue repair (El Morr et al., 2024; Frede et al., 2022; Wang et al., 2021). Notably, type 3 innate lymphoid cells (ILC3s) represent a critical subset of immune cells predominantly residing in the intestinal mucosa (Horn and Sonnenberg, 2024). These cells secrete interleukin-22 (IL-22), a key cytokine essential for epithelial regeneration and the maintenance of barrier function (Horn and Sonnenberg, 2024). Moreover, the gut microbiota is intricately linked to immune regulation, as microbial components can influence the activation and function of ILC3s (Zhong et al., 2024). The modulation of IL-22 secretion by ILC3s, in conjunction with microbial structures, holds promise as a novel therapeutic mechanism for improving anastomotic healing.

Importantly, intestinal immune responses are tightly intertwined with the gut microbiota. Commensal bacteria and fungi provide critical signals that shape ILC3 differentiation, activation, and cytokine production, including IL-22 secretion (Jain et al., 2021; Pickard et al., 2014). Alterations in microbial composition and metabolic output can profoundly influence immune balance, epithelial regeneration, and wound healing outcomes. Emerging evidence indicates that the gut mycobiome, in addition to bacterial communities, contributes to mucosal homeostasis and tissue repair, yet its role in gastrointestinal anastomotic healing remains largely unexplored.

Based on these observations, we hypothesized that the SIS bio-patch may function not merely as a passive mechanical scaffold, but as an immunoactive biomaterial capable of shaping the local immune–microbial–metabolic microenvironment at the anastomotic site. In this study, we employed a murine gastrointestinal anastomosis model to systematically evaluate the effects of SIS on early anastomotic healing. By integrating immune profiling with bacterial and fungal microbiota analyses and untargeted metabolomics, we aimed to elucidate whether SIS enhances anastomotic repair through modulation of IL-22–producing ILC3s and coordinated remodeling of the intestinal microenvironment.

## 2 Materials and methods

### 2.1 Materials

The SIS bio-patches were purchased from Beijing Biosis Healing Biological Technology Co., Ltd. (China).

### 2.2 Animals

Eight-week-old male wild-type C57BL/6 mice were obtained from the Guangdong Medical Laboratory Animal Center and housed at a controlled temperature (22–23 °C) with a 12-h light/dark cycle. The mice had unrestricted access to a standard diet and water in the pathogen-free facilities of the First Affiliated Hospital Animal Center at Guangdong Pharmaceutical University. The study began after a 6-week adaptation period for feeding. All animal procedures were approved by the Animal Ethics Committee of the First Affiliated Hospital of Guangdong Pharmaceutical University (Approval No. 2024004) and were conducted in accordance with institutional guidelines for animal care and use.

### 2.3 Gastrointestinal anastomosis procedure

Fourteen mice were randomly assigned to either the control group or the SIS bio-patch group (n = 7 per group) and fasted for 18 h before surgery. General anesthesia was induced by intraperitoneal injection of tiletamine–zolazepam (Telazol, VIRBAC) at a dose of 50 mg/kg.

A midline abdominal incision (∼2 cm) was made to expose the gastrointestinal tract. An enterotomy was created at the gastrointestinal junction, followed by *in situ* anastomotic repair using interrupted sutures, with minor modifications based on previously described protocols (Eyarefe and Amid, 2010). In the SIS group, an SIS bio-patch was applied to cover the anastomotic site prior to suturing, whereas mice in the control group underwent anastomosis using sutures alone without patch reinforcement. Intra-abdominal bleeding was absorbed using sterile cotton balls. The abdominal wall was closed in two layers, including continuous suturing of the muscle layer and subcuticular closure of the skin. Postoperative analgesia was provided using meloxicam, and cefuroxime was administered to prevent infection. Mice were fasted for 24 h after surgery and subsequently resumed normal feeding. Intraoperative blood loss was estimated by measuring the weight difference of cotton balls before and after blood absorption.

Five days after surgery, mice were euthanized by intraperitoneal administration of Telazol (200 mg/kg) under deep anesthesia in accordance with AVMA guidelines. Anastomotic intestinal tissues were harvested for histological analysis, immunofluorescence staining, flow cytometry, enzyme-linked immunosorbent assay (ELISA), bacterial 16S rRNA sequencing, fungal internal transcribed spacer (ITS) sequencing, and metabolomics analysis. Peripheral blood samples were also collected for flow cytometric analysis. Postoperative day 5 was selected as a representative early healing time point based on previous studies demonstrating its critical role in immune-mediated epithelial repair and tissue remodeling (Ellison, 1989; Jain et al., 2021; Wang et al., 2021).

### 2.4 Histological analysis

Anastomotic tissues from mice were fixed in 4% PFA overnight, paraffin-embedded, and sectioned for hematoxylin and eosin staining. Tissue pathology was evaluated using a modified scoring system (Bergstrom et al., 2010). Assessments included submucosal edema (0 = no change; 1 = mild; 2 = moderate; 3 = profound), epithelial hyperplasia (0 = no change; 1 = 1–50% increase; 2 = 51–100% increase; 3 = over 100%), epithelial integrity (0 = no change; 1 = up to 10 cells shed; 2 = 11–20 cells shed; 3 = ulceration; 4 = ulceration with severe crypt damage), and immune cell infiltration (0 = none; 1 = mild; 2 = moderate; 3 = severe).

### 2.5 Immunofluorescence

Using established methods (Zhong et al., 2024), deparaffinized anastomotic tissue sections were subjected to heat-induced antigen retrieval and then treated for 30 min with 5% BSA and 0.2% Triton X-100 in PBS at room temperature. Post-blocking, the sections were incubated overnight at 4 °C with primary antibodies targeting ZO1 tight junction (1:100; Abcam, ab96587) and Occludin (1:100; Abcam, ab216327). After extensive PBS washes, the sections were exposed to CY3-labeled secondary antibodies. Nuclei were stained with DAPI, and images were captured using a Pannoramic MIDI fluorescence microscope. Fluorescence intensity was quantified with ImageJ software.

### 2.6 Enzyme-linked immunosorbent assay

Mouse TNF-α, interleukin (IL)-6, IL-17A, and IL-22 concentrations were measured using ELISA MAX™ Deluxe Sets (BioLegend, United States of America) in accordance with the manufacturer’s instructions.

### 2.7 Cell isolation

Anastomotic intestinal tissues from mice were harvested, rinsed with PBS to eliminate fecal matter, and the mesenteric adipose was removed. The intestines were longitudinally opened and incubated in an extraction solution containing RPMI-1640, 2% FBS, 10 mM DTT, and 1 mM EDTA at 37 °C, shaking at 180 rpm for 15 min. The tissues were then washed with PBS, cut into 1 mm pieces, and digested in a solution of RPMI-1640, 2% FBS, 1 mg/mL collagenase type II, and 0.5 mg/mL Dispase at 37 °C with constant shaking at 180 rpm for 45 min. After digestion, the tissues were filtered through a 70 μm strainer, and the supernatant was centrifuged to isolate lamina propria leukocytes.

Mouse peripheral blood mononuclear cells (PBMCs) were isolated by treating whole blood with ACK Lysis Buffer to remove erythrocytes, followed by washing and resuspension in PBS.

### 2.8 Flow cytometry analysis

To characterize subsets of innate lymphoid cells (ILCs) in mice, cell suspensions were stained with antibodies against CD3ε, CD5, CD11b, CD11c, CD19, TER-119, CD45, CD127, GATA-3, T-BET, IL-17A, IL-22 (all sourced from BioLegend), and RORγt (eBioscience). Intranuclear staining utilized a Foxp3 staining kit from eBioscience, according to the manufacturer’s guidelines.

For the identification of Th17 and Treg cells, cells were stained with antibodies targeting CD3, CD4, CD45, Foxp3 (all from BioLegend), and RORγt (eBioscience). To delineate macrophage subsets, staining was performed using antibodies against CD45, CD11b, F4/80, CD86, and CD206 (all from BioLegend). Stained cells were then analyzed using a CytoFlex flow cytometer (Beckman Coulter), with data processed via CytExpert software (Beckman Coulter).

### 2.9 Bacterial and fungal amplicon sequencing and analysis

DNA extraction, sequencing, and analytical procedures were conducted by Shanghai Majorbio Bio-Pharm Technology Co., Ltd. (Shanghai, China) as previously described (Zhong et al., 2023). DNA was extracted from anastomotic intestinal tissues using the E. Z.N.A.® Soil DNA Kit (Omega Bio-tek, Norcross, GA, United States). For bacterial analysis, the V3-V4 regions of the 16S rRNA gene were amplified via polymerase chain reaction (PCR) with primers 338F (ACT​CCT​ACG​GGA​GGC​AGC​AG) and 806R (GGACTACHVGGGTWTCTAAT). For fungal analysis, the Internal Transcribed Spacer region 1 (ITS1) was amplified using primers ITS1F (CTT​GGT​CAT​TTA​GAG​GAA​GTA​A) and ITS2R (GCT​GCG​TTC​TTC​ATC​GAT​GC). Sequencing was performed on an Illumina MiSeq platform, and results were deposited in the NCBI database under accession number PRJNA1149522.

Sequence merging was performed with FLASh software (version 1.2.11) and quality filtering with fastp (version 0.19.6) (Chen et al., 2018; Magoc and Salzberg, 2011). Amplicon sequence variants (ASVs) were generated using DADA2 (Callahan et al., 2016), and taxonomic assignment was completed using QIIME2 and the SILVA 16S rRNA database. All analyses were carried out on the Majorbio Cloud Platform (www.majorbio.com).

### 2.10 Metabolomics analysis

A 50 mg anastomotic intestinal tissue sample was prepared for metabolite extraction using a 400 µL methanol solution (4:1, v/v) with 0.02 mg/mL L-2-chlorophenylalanine as an internal standard. The sample was processed using the Wonbio-96c high-throughput tissue crusher (Shanghai Wanbo Biotechnology Co., Ltd.) at −10 °C and 50 Hz for 6 min, followed by ultrasonication at 40 kHz for 30 min at 5 °C. The sample was then cooled to −20 °C for 30 min to precipitate proteins, followed by centrifugation at 13,000 g at 4 °C for 15 min. The supernatant was transferred to vials for LC-MS/MS analysis. To ensure system consistency and quality control, a pooled QC sample was created by mixing equal volumes from all samples.

LC-MS/MS analysis was conducted on a Thermo UHPLC-Q Exactive HF-X system using an ACQUITY HSS T3 column, with mobile phases of 0.1% formic acid in water (95:5, v/v) and acetonitrile: isopropanol (47.5:47.5, v/v). The flow rate was 0.40 mL/min, and the column temperature was set at 40 °C, with an injection volume of 3 µL. Mass spectrometry was performed in both positive and negative modes under optimized settings including auxiliary gas heating, capillary temperature, gas flow rates, and ion-spray voltage. Data was acquired over a mass range of 70–1,050 m/z using Data Dependent Acquisition mode, with full MS resolution at 60,000 and MS/MS resolution at 7,500.

Data processing was performed with Progenesis QI software, which facilitated baseline filtering, peak identification, retention time correction, and peak alignment. The processed data matrix included sample names, m/z values, retention times, and peak intensities. Metabolite identification was carried out using the HMDB, Metlin, and Majorbio databases, with further analysis conducted on the Majorbio cloud platform.

### 2.11 Statistical analysis

Data were analyzed using GraphPad Prism version 8.0.2 (GraphPad Software Inc., United States), and variables were reported as mean ± standard deviation. The unpaired Student’s t-test or Mann-Whitney U test was employed to compare two groups as applicable. A p-value below 0.05 denoted statistical significance.

## 3 Results

### 3.1 The SIS bio-patch does not reduce intraoperative bleeding or early postoperative mortality

**FIGURE 1:**
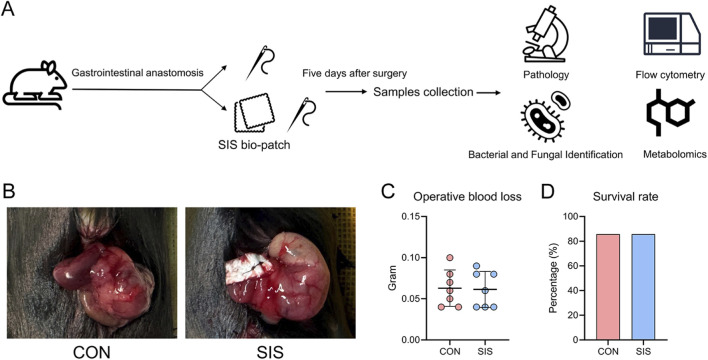
The SIS bio-patch did not reduce bleeding or postoperative mortality in mouse gastrointestinal anastomosis procedures: **(A)** Schematic of the study design; **(B)** Representative image of the gastrointestinal anastomosis; **(C)** Quantitative comparison of intraoperative blood loss between the two groups of mice; **(D)** Percentage of 5-day postoperative survival in the two groups of mice. n = 7 per groups. CON, control; SIS, small intestinal submucosa.

To evaluate the effects of SIS bio-patch reinforcement on gastrointestinal anastomosis outcomes, fourteen mice were randomly assigned to either the control group or the SIS group (n = 7 per group). The experimental workflow and representative images of the anastomosis are shown in Figures 1A,B, respectively. Quantification of intraoperative blood loss revealed no significant difference between the SIS-treated and control groups (0.063 ± 0.022 g vs. 0.061 ± 0.022 g, *P* = 0.906; Figure 1C). In addition, 1 mouse from each group died within the first postoperative day, resulting in an identical survival rate of 85.7% in both groups (Figure 1D). These results indicate that SIS bio-patch application does not significantly affect intraoperative bleeding or early postoperative survival in this mouse model.

### 3.2 The SIS bio-patch attenuates inflammation and enhances mucosal barrier integrity at the anastomotic site

**FIGURE 2:**
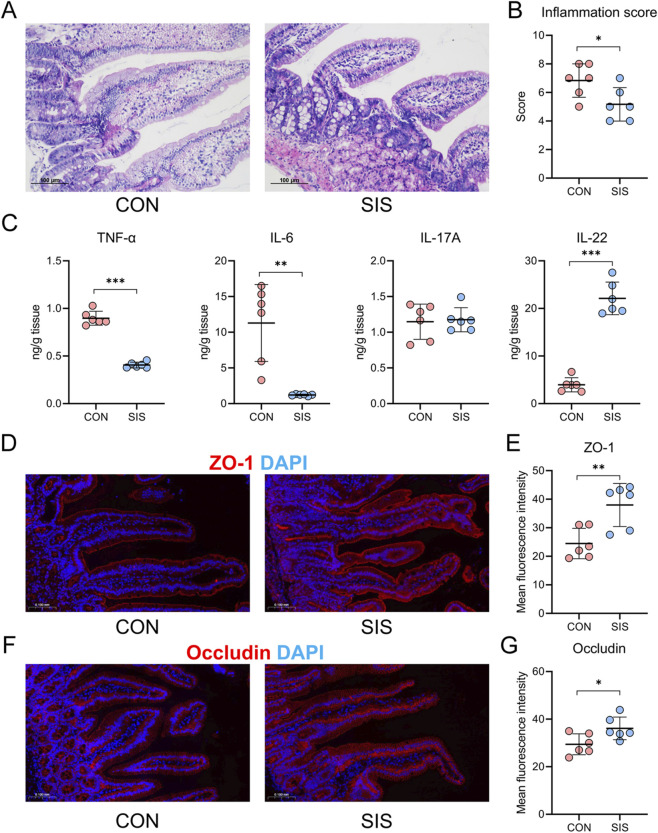
The SIS bio-patch significantly reduced inflammation at the anastomosis site and enhanced intestinal mucosal barrier function: **(A)** Representative histological images of the gastrointestinal anastomosis site for both groups of mice; **(B)** Quantitative comparison of inflammation scores at the anastomosis site; **(C)** Quantitative comparison of cytokine levels in gastrointestinal anastomosis tissues between the two groups; **(D)** Representative ZO-1 immunofluorescence images at the anastomosis site; **(E)** Quantitative comparison of ZO-1 fluorescence at the anastomosis site; **(F)** Representative occludin immunofluorescence images at the anastomosis site; **(G)** Quantitative comparison of occludin fluorescence at the anastomosis site. n = 6 per group. CON, control; SIS, small intestinal submucosa. *p < 0.05; **p < 0.01; ***p < 0.001.

Histological assessment of anastomotic tissues at postoperative day 5 demonstrated markedly reduced submucosal edema and inflammatory cell infiltration in the SIS-treated group compared with controls (Figures 2A,B). Consistently, quantitative histological scoring revealed significantly lower inflammation scores in the SIS group. Analysis of cytokine levels in anastomotic tissues showed that SIS treatment significantly decreased the concentrations of the pro-inflammatory cytokines TNF-α and IL-6, while markedly increasing IL-22 levels (Figure 2C). Immunofluorescence staining further revealed enhanced expression of the tight junction proteins ZO-1 and occludin in the SIS-treated group relative to controls (Figures 2D–G). Together, these findings indicate that SIS bio-patch application alleviates local inflammation and promotes restoration of intestinal epithelial barrier integrity at the anastomotic site.

### 3.3 The SIS bio-patch increases IL-22^+^ ILC3s and modulates the Th17/Treg balance

**FIGURE 3:**
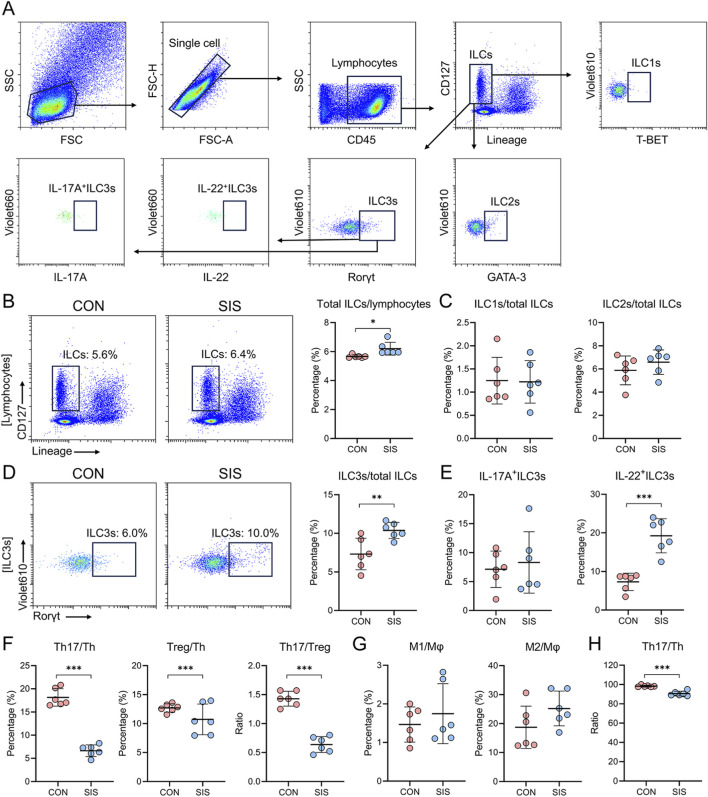
The SIS bio-patch increased levels of IL-22^+^ILC3s at the anastomosis site and regulated the Th17/Treg balance: **(A)** Representative flow cytometric gating strategy for ILC subsets; **(B)** Quantification of total ILCs; **(C)** Proportions of ILC1s/total ILCs and ILC2s/total ILCs; **(D)** Proportions of ILC3s/total ILCs; **(E)** Ratios of IL-17A^+^ILC3s/ILC3s and IL-22^+^ILC3s/ILC3s; **(F)** Ratios of Th17/Th, Treg/Th, and Th17/Treg; **(G)** Ratios of M1/Mφ and M2/Mφ at the gastrointestinal anastomosis site in both groups of mice; **(H)** Quantification of Th17/Th ratios in peripheral blood for both groups of mice. n = 6 per group. CON, control; SIS, small intestinal submucosa. *p < 0.05; **p < 0.01; ***p < 0.001.

Flow cytometric analysis was performed to characterize immune cell populations at the anastomotic site. The gating strategy for innate lymphoid cell (ILC) subsets is shown in Figure 3A. The proportion of total ILCs among lymphocytes was significantly higher in the SIS-treated group than in the control group (Figure 3B). While the relative proportions of ILC1s and ILC2s were not significantly altered (Figure 3C), the proportion of ILC3s was significantly increased in the SIS group (Figure 3D).

Notably, the frequency of IL-22–producing ILC3s was markedly elevated following SIS bio-patch application (Figure 3E). Analysis of adaptive immune subsets revealed a reduction in both Th17 and Treg cells in the SIS group; however, the decrease in Th17 cells was more pronounced, resulting in a significantly lower Th17/Treg ratio compared with controls (Figure 3F). In contrast, no significant differences were observed in the proportions of M1 or M2 macrophages between groups (Figure 3G). Consistent with local immune changes, the Th17/Th ratio in peripheral blood mononuclear cells was also significantly reduced in SIS-treated mice (Figure 3H). These data suggest that SIS bio-patch application selectively enhances IL-22^+^ ILC3 responses while rebalancing adaptive immune subsets at the anastomotic site.

### 3.4 The SIS bio-patch reshapes bacterial community structure and function at the anastomotic site

**FIGURE 4:**
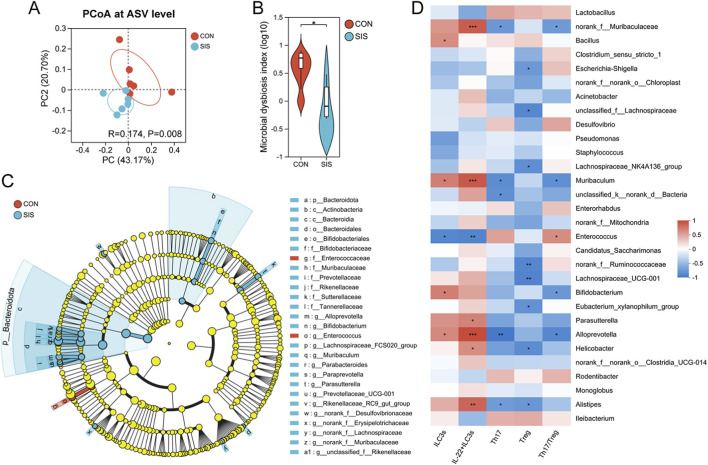
The SIS bio-patch altered the structure and function of bacteria at the anastomotic site: **(A)** Principal Coordinate Analysis (PCoA) illustrating structural differences in the microbiota at the gastrointestinal anastomosis site; **(B)** Microbial dysbiosis index of the microbiota at the gastrointestinal anastomosis site; **(C)** Cladogram based on LEfSe analysis, depicting differential genus abundance in the microbiota at the gastrointestinal anastomosis site; **(D)** Correlation between microbial abundance and immune cell levels at the gastrointestinal anastomosis site in both groups of mice. n = 6 per group. CON, control; SIS, small intestinal submucosa. *p < 0.05.

To determine whether SIS bio-patch application influences the local microbiota, bacterial 16S rRNA gene sequencing was performed on anastomotic tissues. Alpha diversity analysis revealed a trend toward increased microbial richness and diversity in the SIS group, although these differences did not reach statistical significance (Supplementary Figure S1A). Principal Coordinate Analysis (PCoA) demonstrated significant structural differences in the microbiota at the anastomotic sites between mice treated with the SIS bio-patch and those in the control group, accompanied by a marked reduction in the microbial dysbiosis index (Figures 4A,B). The variations in bacterial species and their relative abundances at the anastomotic sites are depicted in Supplementary Figure S1B,C.

Further differential abundance analysis revealed that the SIS bio-patch increased the abundance of beneficial bacteria such as *Bifidobacterium* and *Alloprevotella*, while decreasing that of *Enterococcus* at the anastomotic sites (Figure 4C; Supplementary Figure S1D). Correlation heatmaps indicated that the abundance of *Bifidobacterium*, *Alloprevotella*, and *Muribaculum* on the anastomotic mucosa positively correlated with intestinal ILC3s and IL-22^+^ILC3s, and negatively with the Th17/Treg ratio (Figure 4D), suggesting that the SIS bio-patch may modulate the proportion of mucosal immune cells through the augmentation of these bacteria at the anastomosis.

Predictive functional profiling of the bacterial communities suggested that the SIS bio-patch enhances bacterial functions related to platelet activation, vascular smooth muscle contraction, and protein digestion and absorption, while reducing those associated with *Vibrio cholerae* infection (Supplementary Table S1). These findings imply that the SIS bio-patch may mitigate anastomotic inflammation by modifying the structural and functional dynamics of the associated bacterial communities.

### 3.5 The SIS bio-patch alters fungal community composition at anastomotic sites

**FIGURE 5:**
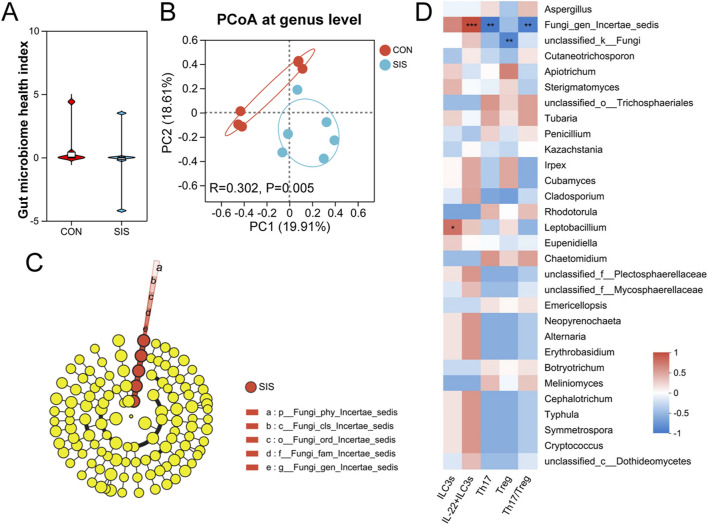
The SIS bio-patch exerted an influence on fungal characteristics at anastomotic sites: **(A)** Gut microbiome health index of fungi at the gastrointestinal anastomosis site; **(B)** Principal Coordinate Analysis (PCoA) showing structural differences in fungal communities at the gastrointestinal anastomosis site; **(C)** Cladogram based on LEfSe analysis, highlighting differential genus abundance of fungi at the gastrointestinal anastomosis site; **(D)** Correlation between fungal abundance and immune cell levels at the gastrointestinal anastomosis site in both groups of mice. n = 6 per group. CON, control; SIS, small intestinal submucosa.

Fungal ITS sequencing was performed to assess changes in the local mycobiome following SIS treatment. While comparative analyses revealed no significant differences in fungal richness, diversity, or health indices between the cohorts, substantial variations in fungal community structure were apparent (Figures 5A,B and Supplementary Figure S2A). The Venn diagram and community bar plot analysis revealed significant disparities in the composition and proportions of fungal species and genera across the mucosal communities of the cohorts (Supplementary Figure S2B,C). Further investigations into genus abundance discrepancies indicated a notable increase in the abundance *Fungi gen.* Incertae sedis within the SIS-treated group. This increase was positively correlated with the levels of intestinal IL-22^+^ILC3s and inversely correlated with Th17 cells and the Th17/Treg ratio (Figures 5C,D and Supplementary Figure S2D). These findings suggest that the SIS bio-patch may modulate inflammation at anastomotic sites by influencing the abundance of *Fungi gen.* Incertae sedis.

### 3.6 The SIS bio-patch modifies metabolic profiles at the anastomotic site

**FIGURE 6:**
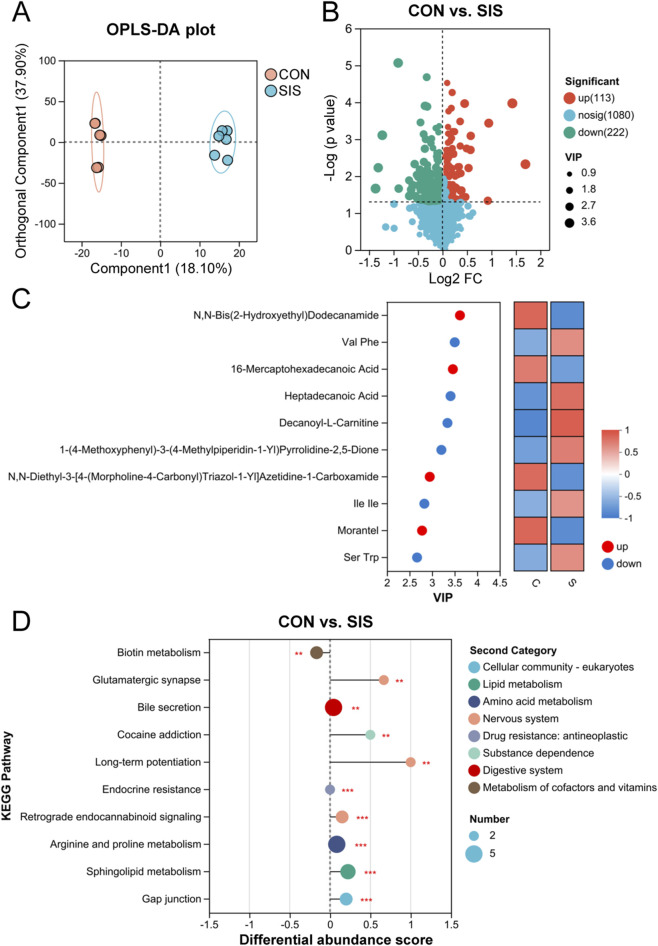
The SIS bio-patch altered the metabolic characteristics of intestinal tissues at anastomotic sites: **(A)** Orthogonal partial least-squares discriminant analysis (OPLS-DA) score plot of metabolites at the gastrointestinal anastomosis site; **(B)** Volcano plot depicting upregulated and downregulated metabolites at the gastrointestinal anastomosis site; **(C)** The top 10 differential metabolites at the gastrointestinal anastomosis site between the two groups of mice; **(D)** KEGG enrichment analysis of differentially abundant metabolites at the gastrointestinal anastomosis site. n = 6 per group. CON, control; SIS, small intestinal submucosa. **p < 0.01; ***p < 0.001.

Untargeted metabolomic analysis revealed clear separation between SIS-treated and control groups based on orthogonal partial least squares discriminant analysis (OPLS-DA) (Figure 6A). Volcano plots revealed that, compared to the SIS-treated group, the control group exhibited a significant increase in the abundance of 113 metabolites and a decrease in 222 metabolites (Figure 6B). The top ten significantly different metabolites between the two groups were identified using the variable importance in projection (VIP) value, as shown in Figure 6C. Notably, metabolites such as N,N-Bis(2-hydroxyethyl)dodecanamide and 16-Mercaptohexadecanoic acid were significantly reduced in the SIS group, while others including Val, Phe, Heptadecanoic Acid, and Decanoyl-L-Carnitine showed significant increases. Correlations between metabolite abundances and immune cell levels at the anastomotic sites are depicted in Supplementary Figure S3. KEGG pathway analysis of differential metabolites indicated a significant enhancement in Biotin metabolism pathways in the SIS group, whereas pathways such as glutamatergic synapse and bile secretion were significantly weakened (Figure 6D). These findings suggest that the SIS bio-patch may alleviate inflammation at anastomotic sites by modifying the metabolic characteristics of mouse intestinal tissues.

## 4 Discussion

In this study, we demonstrate that reinforcement of gastrointestinal anastomoses with an SIS bio-patch significantly improves the local healing microenvironment during the early postoperative phase. Specifically, SIS application attenuated inflammatory responses, enhanced epithelial barrier integrity, increased the abundance of IL-22–producing type 3 innate lymphoid cells, and reshaped bacterial, fungal, and metabolic profiles at the anastomotic site. These findings suggest that SIS functions not only as a mechanical scaffold but also as an immunoactive biomaterial capable of orchestrating coordinated immune–microbial–metabolic interactions to promote gastrointestinal anastomotic repair.

Although SIS is a commercially available biomaterial that has been widely used in tissue repair, its mechanisms of action in gastrointestinal anastomotic healing have remained largely undefined. Previous studies have primarily emphasized the structural reinforcement and biomechanical advantages of SIS, such as its ability to improve early sealing strength and reduce anastomotic leakage (Badylak et al., 2009; Hoeppner et al., 2010). In contrast, the present study provides evidence that SIS exerts biologically active effects on the intestinal microenvironment, extending beyond passive mechanical support. By integrating immune profiling with multi-omics analyses at a critical early healing stage, our work offers mechanistic insight into how SIS modulates local inflammation, immunity, and tissue regeneration.

In clinical practice, several adjunctive approaches have been developed to improve gastrointestinal anastomotic healing, including fibrin sealants, collagen-based dressings, and synthetic or bioabsorbable patches. These materials are primarily designed to enhance mechanical reinforcement, improve early sealing, or provide passive protection at the anastomotic site (Hoeppner et al., 2010; Nordentoft et al., 2007). While such strategies may reduce the risk of early leakage, they generally lack biological activity to actively modulate local immune responses or the intestinal microenvironment. In contrast, small intestinal submucosa represents a biologically derived extracellular matrix scaffold that retains multiple bioactive components capable of interacting with host tissues. Beyond mechanical support, SIS degradation products have been reported to release matrix-bound signals that influence cell migration, differentiation, and immune responses (Badylak et al., 2009). Our findings extend these observations by demonstrating that SIS bio-patch application is associated with enhanced IL-22–producing ILC3 responses, coordinated remodeling of bacterial and fungal communities, and metabolic reprogramming at the anastomotic site. These features distinguish SIS from conventional wound dressings and suggest that its therapeutic potential in gastrointestinal anastomotic healing lies in its ability to integrate structural reinforcement with immunomodulatory and microenvironmental regulation.

A central finding of this study is the marked expansion of IL-22^+^ ILC3s at the anastomotic site following SIS bio-patch application. ILC3-derived IL-22 is a well-established mediator of intestinal epithelial repair, promoting epithelial proliferation, enhancing tight junction integrity, and inducing antimicrobial peptide expression (Araujo et al., 2024; Horn and Sonnenberg, 2024). Consistent with these known functions, increased IL-22 levels in SIS-treated tissues were accompanied by enhanced expression of the tight junction proteins ZO-1 and occludin, as well as reduced levels of pro-inflammatory cytokines TNF-α and IL-6. These coordinated changes suggest that SIS promotes a pro-reparative immune milieu that favors barrier restoration rather than prolonged inflammation.

Beyond innate immunity, SIS application also modulated adaptive immune balance at the anastomotic site. Although both Th17 and Treg populations were reduced, the more pronounced decrease in Th17 cells resulted in a significantly lower Th17/Treg ratio. Given that excessive Th17-driven inflammation has been implicated in impaired intestinal healing and barrier dysfunction, this shift likely contributes to the observed attenuation of local inflammation. Notably, SIS did not significantly alter macrophage polarization in this model, indicating that its immunomodulatory effects in gastrointestinal anastomotic healing may preferentially involve lymphoid rather than myeloid immune pathways. This finding contrasts with reports of SIS-induced M2 macrophage polarization in other tissues, such as peripheral nerve injury models (Zhukauskas et al., 2021), highlighting the tissue-specific nature of SIS–immune interactions.

The beneficial immunological effects of SIS appear to be closely linked to remodeling of the local microbiota. SIS treatment enriched bacterial genera such as *Bifidobacterium* and *Alloprevotella*, which are known to support mucosal immunity and regulate inflammatory responses through microbial metabolites and host–microbe signaling (Groeger et al., 2013; Han et al., 2024). Importantly, the abundance of these bacteria positively correlated with IL-22^+^ ILC3 levels and negatively correlated with the Th17/Treg ratio, suggesting that microbial shifts may act upstream to reinforce SIS-induced immune regulation. These observations are consistent with emerging evidence that microbiota-derived signals critically shape ILC3 function and epithelial repair programs (Pickard et al., 2014).

In addition to bacterial communities, our study highlights a potential role for the intestinal mycobiome in anastomotic healing. SIS application selectively increased the abundance of *Fungi* gen. Incertae sedis, which was positively associated with IL-22^+^ ILC3s and inversely associated with pro-inflammatory immune signatures. Although the precise taxonomy and function of this fungal group remain unclear, prior studies have demonstrated that fungal components can either impair or support mucosal healing depending on context (Jain et al., 2021). Our findings suggest that SIS may promote a fungal community structure that favors immune homeostasis and tissue repair, underscoring the need to consider fungal–immune interactions in gastrointestinal surgical outcomes.

Metabolomic analysis further revealed that SIS bio-patch application reshaped the metabolic landscape of anastomotic tissues. Enrichment of amino acids such as valine and phenylalanine, along with fatty acid–related metabolites and acylcarnitines, is consistent with a metabolic environment supportive of cellular proliferation and tissue remodeling (Eming et al., 2021). Notably, SIS treatment significantly enhanced biotin metabolism, a pathway increasingly recognized as essential for intestinal epithelial homeostasis. Biotin deficiency has been shown to induce intestinal dysbiosis and barrier dysfunction, whereas adequate biotin availability supports epithelial regeneration and mucosal integrity (Erbach et al., 2022; Zempleni and Mock, 2001). These metabolic changes provide an additional mechanistic layer linking SIS application to improved anastomotic healing.

Taken together, the convergent immune, microbial, and metabolic alterations observed in this study strongly suggest that SIS promotes gastrointestinal anastomotic repair by establishing an integrated pro-reparative microenvironment. While causality cannot be definitively established in the present work, the consistency between increased IL-22^+^ ILC3 responses, favorable microbial remodeling, enhanced barrier protein expression, and reduced inflammatory cytokines supports a coordinated biological effect rather than isolated associations.

Several limitations of this study should be acknowledged. First, the investigation focused on an early postoperative time point, and longer-term studies are required to assess sustained immune modulation, microbiota stability, and functional tissue remodeling. Second, this work was conducted in a murine model, and anatomical and physiological differences between mice and humans may influence SIS handling, fixation, and integration in clinical gastrointestinal surgery. In particular, secure attachment of SIS bio-patches to dynamic, peristaltic human intestinal tissue may require optimized suturing techniques or adjunct fixation strategies. Finally, targeted functional experiments will be necessary to establish causal relationships between specific immune cell subsets, microbial taxa, or metabolic pathways and anastomotic healing outcomes.

In conclusion, this study demonstrates that SIS bio-patch reinforcement enhances gastrointestinal anastomotic healing through integrated modulation of immune responses, microbial communities, and metabolic pathways. By identifying IL-22^+^ ILC3s as a central immunological node linking SIS application to epithelial repair, our findings provide mechanistic support for the clinical use of SIS in gastrointestinal surgery and inform the future development of multifunctional biomaterials designed to actively promote mucosal healing.

## Data Availability

The datasets presented in this study can be found in online repositories. The names of the repository/repositories and accession number(s) can be found below: https://www.ncbi.nlm.nih.gov/, PRJNA1149522.

## References

[B1] Araujo L. P. Edwards M. Irie K. Huang Y. Kawano Y. Tran A. (2024). Context-dependent role of group 3 innate lymphoid cells in mucosal protection. Sci. Immunol. 9 (98), eade7530. 10.1126/sciimmunol.ade7530 39151019 PMC11586228

[B2] Badylak S. F. Freytes D. O. Gilbert T. W. (2009). Extracellular matrix as a biological scaffold material: structure and function. Acta Biomater. 5 (1), 1–13. 10.1016/j.actbio.2008.09.013 18938117

[B3] Bergstrom K. S. Kissoon-Singh V. Gibson D. L. Ma C. Montero M. Sham H. P. (2010). Muc2 protects against lethal infectious colitis by disassociating pathogenic and commensal bacteria from the colonic mucosa. PLoS Pathog. 6 (5), e1000902. 10.1371/journal.ppat.1000902 20485566 PMC2869315

[B4] Callahan B. J. McMurdie P. J. Rosen M. J. Han A. W. Johnson A. J. Holmes S. P. (2016). DADA2: high-resolution sample inference from illumina amplicon data. Nat. Methods 13 (7), 581–583. 10.1038/nmeth.3869 27214047 PMC4927377

[B5] Chen S. Zhou Y. Chen Y. Gu J. (2018). Fastp: an ultra-fast all-in-one FASTQ preprocessor. Bioinformatics 34 (17), i884–i890. 10.1093/bioinformatics/bty560 30423086 PMC6129281

[B6] El Morr Y. Furstenheim M. Mestdagh M. Franciszkiewicz K. Salou M. Morvan C. (2024). MAIT cells monitor intestinal dysbiosis and contribute to host protection during colitis. Sci. Immunol. 9 (96), eadi8954. 10.1126/sciimmunol.adi8954 38905325 PMC7616241

[B7] Eming S. A. Murray P. J. Pearce E. J. (2021). Metabolic orchestration of the wound healing response. Cell Metab. 33 (9), 1726–1743. 10.1016/j.cmet.2021.07.017 34384520

[B30] Ellison G. W. (1989). Wound healing in the gastrointestinal tract. Semin. Vet. Med. Surg. Small Anim. 4 (4), 287–293. 2697061

[B8] Erbach J. Bonn F. Diesner M. Arnold A. Stein J. Schroder O. (2022). Relevance of biotin deficiency in patients with inflammatory bowel disease and utility of serum 3 hydroxyisovaleryl carnitine as a practical everyday marker. J. Clin. Med. 11 (4), 1118. 10.3390/jcm11041118 35207391 PMC8877558

[B9] Eyarefe O. D. Amid S. A. (2010). Small bowel wall response to enterotomy closure with polypropylene and polyglactin 910 using simple interrupted suture pattern in rats. Int. J. Animal Veterinary Adv. 2 (3), 72–75.

[B10] Frede A. Czarnewski P. Monasterio G. Tripathi K. P. Bejarano D. A. Ramirez Flores R. O. (2022). B cell expansion hinders the stroma-epithelium regenerative cross talk during mucosal healing. Immunity 55 (12), 2336–2351. 10.1016/j.immuni.2022.11.002 36462502

[B11] Groeger D. O'Mahony L. Murphy E. F. Bourke J. F. Dinan T. G. Kiely B. (2013). Bifidobacterium infantis 35624 modulates host inflammatory processes beyond the gut. Gut Microbes 4 (4), 325–339. 10.4161/gmic.25487 23842110 PMC3744517

[B12] Han B. Shi L. Bao M. Y. Yu F. L. Zhang Y. Lu X. Y. (2024). Dietary ellagic acid therapy for CNS autoimmunity: targeting on Alloprevotella rava and propionate metabolism. Microbiome 12 (1), 114. 10.1186/s40168-024-01819-8 38915127 PMC11194905

[B13] Hoeppner J. Wassmuth B. Marjanovic G. Timme S. Hopt U. T. Keck T. (2010). Anastomotic sealing by extracellular matrices (ECM) improves healing of colonic anastomoses in the critical early phase. J. Gastrointest. Surg. 14 (6), 977–986. 10.1007/s11605-010-1191-1 20354808

[B14] Horn V. Sonnenberg G. F. (2024). Group 3 innate lymphoid cells in intestinal health and disease. Nat. Rev. Gastroenterol. Hepatol. 21 (6), 428–443. 10.1038/s41575-024-00906-3 38467885 PMC11144103

[B15] Jain U. Ver Heul A. M. Xiong S. Gregory M. H. Demers E. G. Kern J. T. (2021). Debaryomyces is enriched in crohn's disease intestinal tissue and impairs healing in mice. Science 371 (6534), 1154–1159. 10.1126/science.abd0919 33707263 PMC10114606

[B16] Li X. Hu X. Fu C. Han L. Xie M. Ouyang S. (2023). Efficacy and safety of one anastomosis gastric bypass Versus Roux-en-Y gastric bypass for obesity: a meta-analysis and systematic review. Obes. Surg. 33 (2), 611–622. 10.1007/s11695-022-06401-5 36564618 PMC9889439

[B17] Magoc T. Salzberg S. L. (2011). FLASH: fast length adjustment of short reads to improve genome assemblies. Bioinformatics 27 (21), 2957–2963. 10.1093/bioinformatics/btr507 21903629 PMC3198573

[B18] Marrache M. K. Itani M. I. Farha J. Fayad L. Sharara S. L. Kalloo A. N. (2021). Endoscopic gastrointestinal anastomosis: a review of established techniques. Gastrointest. Endosc. 93 (1), 34–46. 10.1016/j.gie.2020.06.057 32593687

[B19] Nordentoft T. Romer J. Sorensen M. (2007). Sealing of gastrointestinal anastomoses with a fibrin glue-coated collagen patch: a safety study. J. Invest Surg. 20 (6), 363–369. 10.1080/08941930701772173 18097878

[B20] Pickard J. M. Maurice C. F. Kinnebrew M. A. Abt M. C. Schenten D. Golovkina T. V. (2014). Rapid fucosylation of intestinal epithelium sustains host-commensal symbiosis in sickness. Nature 514 (7524), 638–641. 10.1038/nature13823 25274297 PMC4214913

[B21] Velotti N. Vitiello A. Berardi G. Di Lauro K. Musella M. (2021). Roux-en-Y gastric bypass versus one anastomosis-mini gastric bypass as a rescue procedure following failed restrictive bariatric surgery. A systematic review of literature with metanalysis. Updat. Surg. 73 (2), 639–647. 10.1007/s13304-020-00938-9 33606148

[B22] Wang X. Cai J. Lin B. Ma M. Tao Y. Zhou Y. (2021). GPR34-mediated sensing of lysophosphatidylserine released by apoptotic neutrophils activates type 3 innate lymphoid cells to mediate tissue repair. Immunity 54 (6), 1123–1136 e8. 10.1016/j.immuni.2021.05.007 34107271

[B23] Ying X. Yu C. Yang W. Ye L. Sun R. Gu T. (2024). The transformation of multifunctional bio-patch to hydrogel on skin wounds for efficient scarless wound healing. Mater Today Bio 24, 100901. 10.1016/j.mtbio.2023.100901 38188643 PMC10770564

[B24] Zempleni J. Mock D. M. (2001). Biotin homeostasis during the cell cycle. Nutr. Res. Rev. 14 (1), 45–64. 10.1079/NRR200117 19087416

[B25] Zhang N. Huang Y. Wei P. Sun L. Jing W. Xue Y. (2023). Killing two birds with one stone: a therapeutic copper-loaded bio-patch promoted abdominal wall repair via VEGF pathway. Mater Today Bio 22, 100785. 10.1016/j.mtbio.2023.100785 37680583 PMC10480776

[B26] Zhao Y. Peng H. Sun L. Tong J. Cui C. Bai Z. (2024). The application of small intestinal submucosa in tissue regeneration. Mater Today Bio 26, 101032. 10.1016/j.mtbio.2024.101032 38533376 PMC10963656

[B27] Zhong H. J. Wang S. Q. Zhang R. X. Zhuang Y. P. Li L. Yi S. Z. (2023). Supplementation with high-GABA-producing Lactobacillus plantarum L5 ameliorates essential tremor triggered by decreased gut bacteria-derived GABA. Transl. Neurodegener. 12 (1), 58. 10.1186/s40035-023-00391-9 38093327 PMC10717605

[B28] Zhong H. J. Zhuang Y. P. Xie X. Song J. Y. Wang S. Q. Wu L. (2024). Washed microbiota transplantation promotes homing of group 3 innate lymphoid cells to the liver via the CXCL16/CXCR6 axis: a potential treatment for metabolic-associated fatty liver disease. Gut Microbes 16 (1), 2372881. 10.1080/19490976.2024.2372881 38940400 PMC11216104

[B29] Zhukauskas R. Fischer D. N. Deister C. Alsmadi N. Z. Mercer D. (2021). A comparative study of porcine small intestine submucosa and cross-linked bovine type I collagen as a nerve conduit. J. Hand Surg. Glob. Online 3 (5), 282–288. 10.1016/j.jhsg.2021.06.006 35415568 PMC8991869

