# Supplementary material for: Enhancement of gastrointestinal anastomosis healing via a small intestinal submucosa bio-patch: modulating IL-22 secretion by type 3 innate lymphoid cells and microbial structures
Source: Front Bioeng Biotechnol. 2026 Feb 13;14:1752619. doi: 10.3389/fbioe.2026.1752619 (PMC12946001; doi:10.3389/fbioe.2026.1752619)
Supplement: Supplementary file 1 [file Supplementaryfile1.docx]

Supplementary Material

# Supplementary Figures


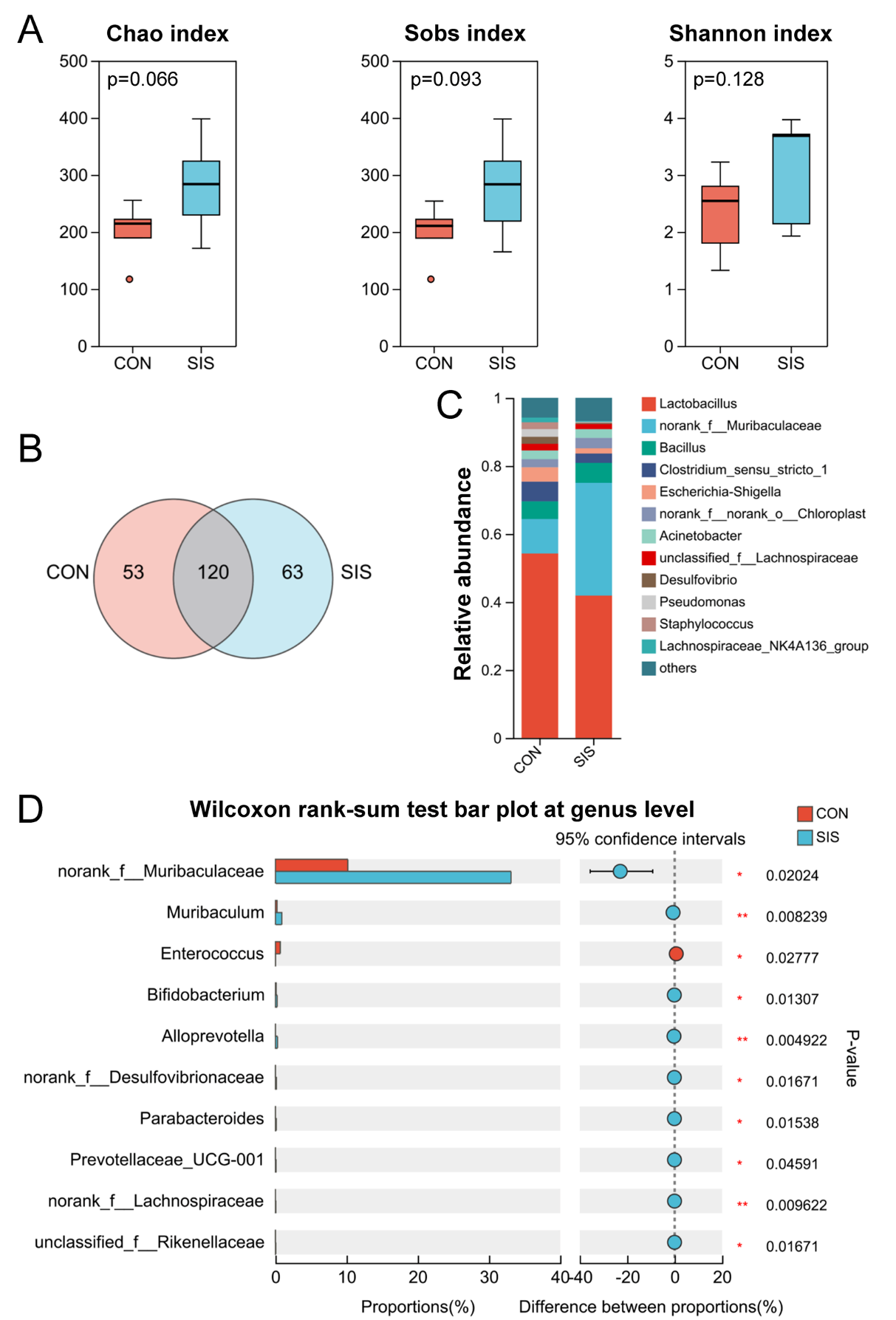


**Supplementary Figure 1.** **The influence of the SIS bio-patch on the microbiota in intestinal tissues at anastomotic sites: (A)** Box plot showing the alpha diversity indices (Chao, Sobs, and Shannon) of the microbiota at the gastrointestinal anastomosis site in both mouse groups; **(B)** Venn diagram illustrating the genus feature distribution at the gastrointestinal anastomosis site in both mouse groups; **(C)** Genus-level distribution of the microbiota at the gastrointestinal anastomosis site; **(D)** Bar chart depicting the relative abundances of the top 10 differentially abundant genera at the gastrointestinal anastomosis site between the two groups of mice. n = 6 per group. CON, control; SIS, small intestinal submucosa. *p < 0.05; **p < 0.01.

**
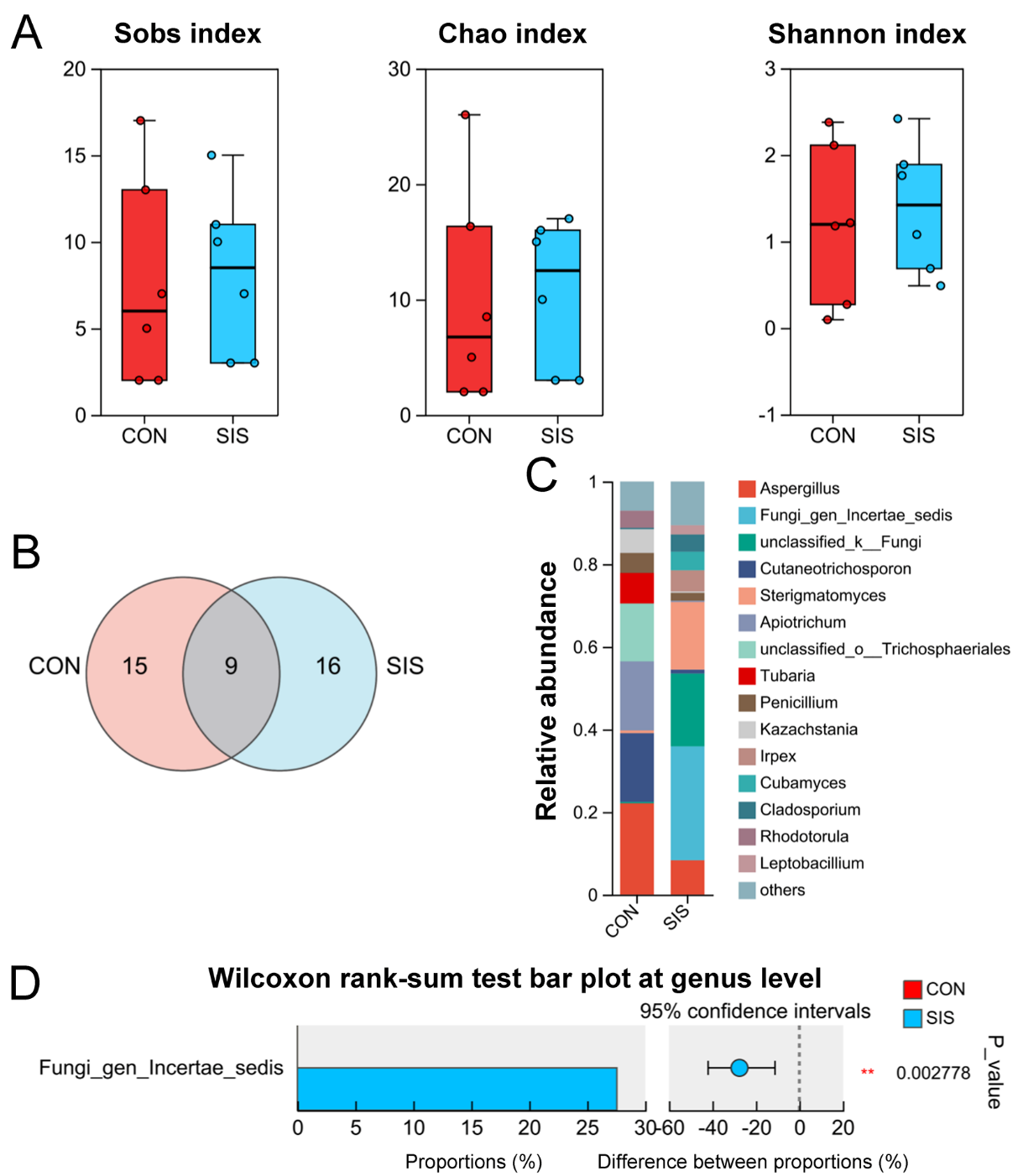
**

**Supplementary Figure 2. The influence of the SIS bio-patch on fungi in intestinal tissues at anastomotic sites: (A)** Box plot showing the alpha diversity indices (Sobs, Chao, and Shannon) of fungi at the gastrointestinal anastomosis site in both mouse groups; **(B)** Venn diagram illustrating the fungal feature distribution at the gastrointestinal anastomosis site in both mouse groups; **(C)** Genus-level distribution of fungi at the gastrointestinal anastomosis site; **(D)** Bar chart depicting the relative abundances of the top 10 differentially abundant fungi at the gastrointestinal anastomosis site between the two groups of mice. n = 6 per group. CON, control; SIS, small intestinal submucosa. **p < 0.01.

**
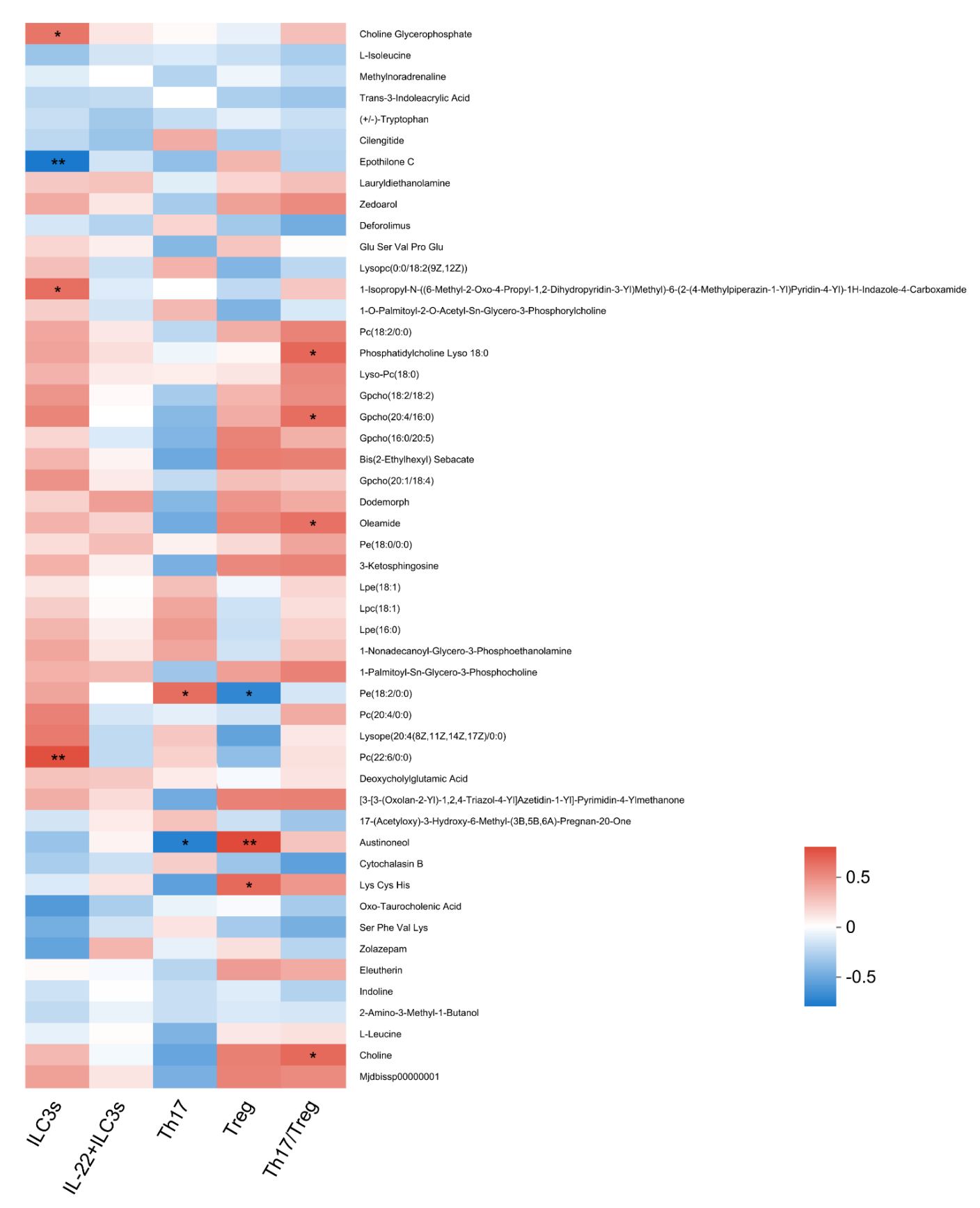
**

**Supplementary Figure 3. The relationship between metabolite levels and immune cell levels in intestinal tissues at anastomotic sites.** *p < 0.05; **p < 0.01.

# Supplementary Table

**Supplementary Table 1. Bacterial Functional Differences Analyzed by PICRUSt2**

| Name | Fold Change(CON/SIS) | P-value |
| --- | --- | --- |
| Oxytocin signaling pathway | 0.019920477 | 0.02024 |
| Oocyte meiosis | 0.020719853 | 0.02024 |
| Long-term potentiation | 0.020719853 | 0.02024 |
| cGMP-PKG signaling pathway | 0.020719853 | 0.02024 |
| Cellular senescence | 0.020719853 | 0.02024 |
| Glycosphingolipid biosynthesis - lacto and neolacto series | 0.021602787 | 0.008239 |
| Adrenergic signaling in cardiomyocytes | 0.023654391 | 0.02024 |
| Platelet activation | 0.024653573 | 0.03064 |
| Insulin secretion | 0.02782818 | 0.02024 |
| Apelin signaling pathway | 0.02782818 | 0.02024 |
| Circadian entrainment | 0.02782818 | 0.02024 |
| Inflammatory mediator regulation of TRP channels | 0.045248823 | 0.03064 |
| Hippo signaling pathway | 0.045248823 | 0.03064 |
| Vascular smooth muscle contraction | 0.045248823 | 0.03064 |
| Arrhythmogenic right ventricular cardiomyopathy (ARVC) | 0.083947417 | 0.04533 |
| Protein digestion and absorption | 0.258401615 | 0.02024 |
| Glycosphingolipid biosynthesis - ganglio series | 0.265800712 | 0.02024 |
| Proteasome | 0.305442177 | 0.04533 |
| Various types of N-glycan biosynthesis | 0.308876365 | 0.03064 |
| Lysosome | 0.309839357 | 0.02024 |
| Flavone and flavonol biosynthesis | 0.395103939 | 0.04533 |
| Other glycan degradation | 0.635751295 | 0.04533 |
| Cyanoamino acid metabolism | 0.853400735 | 0.03064 |
| Protein processing in endoplasmic reticulum | 0.907271702 | 0.04533 |
| Vibrio cholerae infection | 27.27272727 | 0.04533 |
